# Effect of Aerobic Training on Heart Rate Recovery in Patients with Established Heart Disease; a Systematic Review

**DOI:** 10.1371/journal.pone.0083907

**Published:** 2013-12-18

**Authors:** Johan A. Snoek, Sietske van Berkel, Nico van Meeteren, Frank J. G. Backx, Hein A. M. Daanen

**Affiliations:** 1 Sports Medicine Department, Isala, Zwolle, The Netherlands; 2 TNO Healthy Living, Leiden, The Netherlands; 3 Centre for Care Technology Research, Maastricht, The Netherlands; 4 Rehabilitation, Nursing science and Sports Department, UMC Utrecht, Utrecht, The Netherlands; 5 MOVE Research Institute Amsterdam & Faculty of Human Movement Sciences, VU University, Amsterdam, The Netherlands; University of Milan, Italy

## Abstract

**Background:**

Although a delayed decrease in heart rate during the first minute after graded exercise has been identified as a powerful predictor of overall mortality in cardiac patients, the potential to influence this risk factor by aerobic training remains to be proven.

**Objective:**

The aim was to study the relationship between aerobic training and Heart Rate Recovery (HRR) in patients with established heart disease.

**Methods:**

(Quasi) randomized clinical trials on aerobic exercise training in adults with established heart disease were identified through electronic database and reference screening. Two reviewers extracted data and assessed the risk of bias and therapeutic validity. Methodological validity was evaluated using an adapted version of the Cochrane Collaboration’s tool for assessing risk of bias and the therapeutic validity of the interventions was assessed with a nine-itemed, expert-based rating scale (CONTENT). Scores range from 0 to 9 (score ≥ 6 reflecting therapeutic validity).

**Results:**

Of the 384 articles retrieved, 8 studies (449 patients) were included. Three of the included studies demonstrated adequate therapeutic validity and five demonstrated low risk of bias. Two studies showed both adequate therapeutic validity and a low risk of bias. For cardiac patients aerobic exercise training was associated with more improvement in HRR compared to usual care.

**Conclusion:**

The present systematic review shows a level 1A evidence that aerobic training increases HRR in patients with established heart disease.

## Introduction

Exercise based rehabilitation has proven its value in reducing morbidity and mortality in patients with established heart disease [1-5]. The cardiovascular benefits of exercise training are multifactorial and include important local and systemic effects on skeletal muscle, the peripheral vasculature, the myocardium and the autonomic nervous system. Both parasympathetic and sympathetic tone have been shown to respectively in and decrease in humans and animals [6,7]. One of the more short term modulations of the autonomic nervous system seems to be the Heart Rate Recovery (HRR) [8]. HRR can be defined as the rate at which heart rate declines, usually within minutes after the cessation of physical exercise [9,10]. A delayed decrease in heart rate during the first minute after graded exercise is a powerful predictor of overall mortality in both patients with and without heart disease, independent of workload, the presence or absence of myocardial perfusion defects, and changes in heart rate during exercise [11].

Whether HRR may also serve as a powerful and convenient instrument to monitor improvement in training status during exercise based rehabilitation of patients with established heart disease remains, to our knowledge, to be proven. Therefore the aim of this study was to conduct a systematic review on the effect of aerobic training on HRR in patients with established heart disease.

## Methods

### Data sources

A search was performed in the following electronic databases from start to July 2012: MEDLINE (accessed by PubMed), Cochrane Central Register of Controlled Trials, EMBASE, and Scopus. In addition, we manually searched the references of published studies. The initial search was not limited by language and comprised the terms ‘Heart Rate Recovery’, ‘Exercise’ OR ‘Training’ AND ‘Heart Disease’. The complete search strategy used for the different databases is shown in Table S1. This systematic review is reported in accordance with the Preferred Reporting Items for Systematic Reviews and Meta-Analyses (PRISMA) statement (Checklist S1) [12,13]. 

### Study selection: In- and exclusion criteria

Articles published before July 2012 in generally accessible, English-language, peer-reviewed scientific journals were assessed as suitable. Inclusion was based on:

- Articles being full text randomised or quasi-randomised (methods of allocating participants to a treatment which are not strictly random e.g. date of birth, hospital record number or alternation) controlled trials;
- HRR being a dependent variable: the method to determine HRR was not considered as an in- or exclusion criterion;
- The duration of the therapeutic physical training being at least 2 weeks with a pre and post measurement of HRR (The type of training was not considered as an in- or exclusion criterion);
- The control group receiving no exercise therapy or usual care;
- The selected subjects being patients with established heart disease (e.g. STEMI (ST Elevated Myocardial Infarction), CABG (Coronary Artery Bypass Graft), CAD (Coronary artery disease) and AMI (Acute Myocardial Infarction).

Exclusion criteria:

- Patients < 18 years.

### Data Extraction

Two reviewers (S.v.B. and J.A.S.) independently extracted the following information from each eligible publication: year of publication, trial design, study population, number of subjects, type of exercise test, method to determine HRR, and type, duration and intensity of the exercise intervention. Any disagreements about the extracted data were solved by a third reviewer (H.D). In case of missing data, the corresponding author of the included study was contacted.

### Assessment of methodological (risk of bias) validity

Two reviewers (S.v.B. and J.A.S.) independently assessed the methodological validity (risk of bias) of the studies. This was scored using the adapted version of the Cochrane Collaboration’s tool [14]. This adapted tool reviews five domains, with 11 items in total (see Table S2). Each item is rated as ‘yes’, ‘no’, or ‘unsure’. Studies fulfilling six or more items were regarded as having a low risk of bias [15]. The strength of agreement between the two reviewers was measured by Cohen’s k coefficient (95%-confidence intervals), with k= 0.41–0.60 indicating moderate agreement, k= 0.61–0.80 representing good agreement, and k= 0.81 representing very good agreement [16].

### Assessment of therapeutic validity

To assess the therapeutic validity of the different exercise programmes we used the CONTENT scale composed and described by Hoogeboom et al.. They followed the method described by Yates et al [17] to form a Delphi panel. This panel subsequently defined in four rounds a workable 9 item rating scale for the therapeutic validity of exercise programmes. Each item was rated as ‘yes’ or ‘no’. Studies with six or more points out of nine were regarded as being of high therapeutic quality. The strength of agreement between the two reviewers was measured by Cohen’s k coefficient (95%-confidence intervals), with k= 0.41–0.60 indicating moderate agreement, k= 0.61–0.80 representing good agreement, and k= 0.81 representing very good agreement [16].

## Results

### Description of search

**Figure 1:**
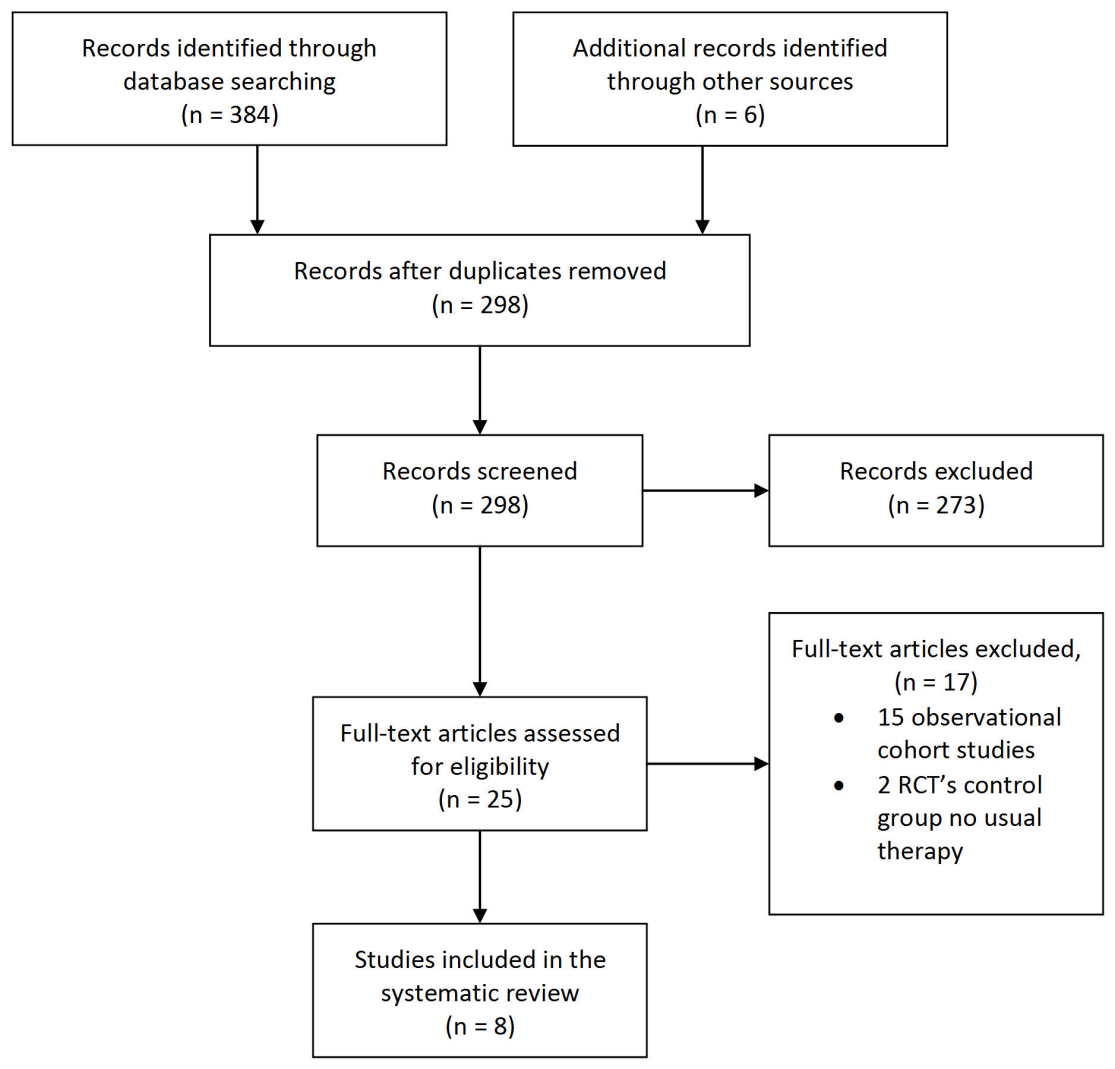
Description of search. Selection of trials investigating heart rate recovery.

We identified a total of 384 records in the initial search and removed 92 duplicate publications. Six records were added through searches in references. We excluded 273 non-relevant records based on title or abstract screening. Full-text articles were retrieved for 25 publications and assessed for eligibility (Figure 1). Four randomized controlled trials and four quasi-randomized controlled trials met the eligibility criteria (Table 1) [18-25]. Giallauria et al. investigated the effect of cycling three times a week with an intensity of 60-70% peak VO_2_ in 37 STEMI patients with 38 controls. Legramante et al. trained 43 subjects with CABG on a bicycle for thirty minutes two times a day during six weeks with an intensity of 75-85 % of maximal heart rate. Moholdt et al. studied the effect of training on a treadmill versus exercises as walking, jogging, lunges and squats on 59 subjects with an AMI with 30 controls during 12 weeks. Twelve patients with chronic heart failure were compared with 12 controls by Myers et al. during eight weeks of cycling and walking training vs usual care. In 2012 Ribeiro et al. investigated the effect of aerobic exercise training on 20 patients with CAD compared to 18 patients with usual care. Tsai et al. trained 15 CABG patients with walking and running training during 12 weeks three times a week and compared them with 15 controls. Wu et al. made three groups of 18 patients with CABG and compared a cardiac rehabilitation program with home based exercises and a group with no exercises during twelve weeks. Finally twenty-seven patients with an AMI were trained by Zeng et al. for twenty-six weeks with cycling training three times a week on the anaerobic threshold for thirty minutes. Table S3 shows the studies not included in the review (15 prospective or retrospective cohort studies and 2 RCT’s in which the control group received a different therapy than usual care or no therapy). 

**Table 1:** Overview of (quasi) Randomized Controlled Trials included in the review.

Author	Study population	Groups	Participants (n)per group	Stress test	Measure-ment HRR	Type of training	Frequency + length of training	Intensity of training	Length of rehab.	Change HRR (60 sec)
Giallauria *et al* (2011)	STEMI	2	37,38	Bicycle	60 sec	Cycling	E: 3 x p.w. 40 min C: untrained	60-70 % peak VO_2_	26 wk	E: 13 → 20* C: 14 → 12*
Legramante *et al* (2007)	CABG	2	43,39	Bicycle	60 sec + other	Cycling	E: 6 x p.w. 2 x p.d 30 min C: untrained	75-85 % HR max	2 wk	E: 8 → 12 C: 7 → 10
Moholdt *et al* (2011)	AMI	2	59,30	Treadmill	60 sec	Treadmill vs W+J+L+S	E: 3 x p.w. 38 min C: 3 x p.w. 60 min	E: 85-95 % HR max C: Vigorous exercise	12 wk	E: 31 → 33 C: 33 → 34
Myers *et al* (2007)	CHF	2	12,12	Bicycle	60 sec + other	Cycling Walking	E: 4 x p.w. 45 min + 2 x p.d. 60 min C: usual care	E: 60-80 % HR reserve	8 wk	E: 8 → 14 C: 9 → 10
Ribeiro *et al* (2012)	CAD	2	20,18	Treadmill	60 sec	Aerobic exercise	E: 3 x p.w. 55 min C: usual care	65-75 % HR max	8 wk	E: 20 → 24* C: 26 → 26*
Tsai *et al* (2005)	CABG	2	15,15	Bicycle	60 sec	Cycling; Walking	E: 3 x p.w. 30-40 min C: No cardiac rehabilitation	60-80 % HR max	12 wk	E: 4 → 16* C: 5 → 11*
Wu *et al* (2006)	CABG	3	18,18,18	Bicycle	60 sec	Cycling; Treadmill	E: 3 x p.w. 50-80 min Eh: 3 x p.w. 50-80 min C: No exercise	60-85 % HR max	12 wk	E: 9 → 19* Eh: 8 → 16 C: 9 → 14*
Zeng *et al* (2008)	AMI	2	27,30	Bicycle	60 sec	Cycling	E: 3 x p.w. 30 min C: No exercise	AT	26 wk	E: 12 → 17* C: 12 → 12*

Abbreviations: n (number); Change HRR (Change in Heart Rate Recovery from begin- to endpoint); STEMI (ST Elevated Myocardial Infarction); CABG (Coronary Artery Bypass Graft); AMI (Acute Myocardial Infarction);W+J+L+S (Walking, Jogging, Lunges, Squats); CHF (Chronic Heart Failure); CAD (Coronary Artery Disease); E (Exercise group); Eh (Exercise at home group); C (Control group); VO_2_ (oxygen uptake); HR (Heart Rate); AT (Anaerobic Threshold) * Significant (p<0.05) between group difference

### Risk of bias

Table 2 shows the methodological quality assessment of individual studies. For one study the corresponding author was contacted to resolve ambiguity in data. The initial agreement of the reviewers on the total risk of bias assessment was 88% (77 of 88 items), and Cohen’s Kappa (95% CI) was 0.79 (0.73–0.85). All disagreements were resolved in a consensus meeting. Three studies were assessed as having a high risk of bias and five studies were assessed as having a low risk of bias. The most prevalent limitations were found in items about blinding (patient, care provider, outcome assessor), allocation concealment and compliance. 

**Table 2:** Assessment of risk of bias per individual study per scale item.

				Blinding								
Study	Adequate randomi-sation	Allocation concealed	Patient	Care provider	Outcome assessor	Drop-out rate described	Intention to treat analysis	Groups similar at baseline	Co inter-ventions avoided	Compliance acceptable	Timing of outcome assessment similar	Total score
Giallauria *et al* (2011)	Yes	Unsure	No	Unsure	Yes	Yes	Yes	Yes	No	Yes	Yes	7 (64%)
Legramente *et al* (2007)	Unsure	Unsure	No	Unsure	Unsure	Yes	Yes	Yes	Yes	Unsure	Yes	5 (46%)
Moholdt *et al* (2011)	Yes	Yes	No	Unsure	Unsure	Yes	Yes	Yes	Yes	Yes	Yes	8 (73%)
Myers *et al* (2007)	Unsure	Unsure	No	Unsure	Unsure	Yes	Yes	Yes	No	Unsure	Yes	4 (36%)
Ribeiro *et al* (2012)	Yes	Yes	No	Unsure	Unsure	Yes	Yes	No	Yes	Unsure	Yes	6 (55%)
Tsai *et al* (2005)	Yes	Yes	No	Unsure	Unsure	Yes	Yes	Yes	Yes	Unsure	Yes	7 (64%)
Wu *et al* (2006)	Yes	Unsure	Yes	Unsure	Yes	Yes	Yes	Yes	Yes	Unsure	Yes	8 (73%)
Zeng *et al* (2008)	No	No	No	Unsure	Unsure	Yes	Yes	Yes	Yes	Unsure	Yes	5 (46%)
Total score	5 (63%)	3 (38%)	1 13%)	0 (0%)	2 (25%)	8 (100%)	8 (100%)	7 (88%)	6 (75%)	2 (25%)	8 (100%)	

### Therapeutical validity

Table 3 shows the therapeutic validity assessment score per individual study as assessed using the CONTENT scale. Cohen’s kappa revealed a moderate agreement between the two reviewers of 0.69 (0.60–0.78); absolute agreement was found in 61 out of 72 items (85%). The item ‘‘Was the therapeutic exercise monitored and adjusted when considered necessary?’’ had the lowest agreement between the reviewers. All disagreements were resolved in a consensus meeting. Three of the eight studies could be labelled as being therapeutically valid according to the cut-off score of six or higher. Both therapeutic validity and methodological validity scores are presented in Table 4.

**Table 3:** Assessment of therapeutic validity per individual study per scale item (CONTENT scale).

	Patient eligibility			Rationale			Content				
Study	Described	Adequate	Setting and therapist	Study	Intervention		Intensity	Monitored	Personalized	Adherence	Total score
Giallauria *et al* (2011)	yes	yes	no	yes		no	yes	no	yes	yes	6 (66%)
Legramente *et al* (2007)	yes	no	no	yes		no	yes	no	no	no	3 (33%)
Moholdt *et al* (2011)	yes	yes	no	yes		no	yes	no	no	yes	5 (55%)
Myers *et al* (2007)	yes	yes	yes	yes		no	yes	no	yes	no	6 (66%)
Ribeiro *et al* (2012)	yes	yes	no	yes		yes	yes	no	no	no	5 (55%)
Tsai *et al* (2005)	no	yes	no	yes		no	yes	no	no	no	3 (33%)
Wu *et al* (2006)	yes	yes	yes	yes		no	yes	no	yes	no	6 (66%)
Zeng *et al* (2008)	yes	yes	no	yes		no	no	no	no	no	3 (33%)
Total score	7 (88%)	7 (88%)	2 (25%)	8 (100%)		1 (13%)	7 (88%)	0 (0%)	3 (38%)	2 (25%)	

**Table 4:** Methodological and therapeutic validity scores per study.

**Study**	**Methodological validity (0-11)**	**Therapeutical validity (0-9)**
Giallauria et al. (2011)*	7 (64%)**	6 (66%)**
Legramante *et al*. (2007)	5 (46%)	3 (33%)
Moholdt *et al*. (2011)	8 (73%)	5 (55%)
Myers *et al*. (2007)	4 (36%)	6 (66%)
Ribeiro *et al*. (2012)*	6 (55%)	5 (55%)
Tsai *et al*. (2005)*	7 (64%)	3 (33%)
Wu *et al*. (2006)*	8 (73%)**	6 (66%)**
Zeng *et al*. (2008)*	5 (46%)	3 (33%)

^*^ Significant (p<0.05) between group difference

^**^ Low risk of bias and a high therapeutical validity

### Association between exercise intervention and HRR

Five of the eight studies showed a significant between group difference of HRR after exercise (Table 4). Four of these five studies showed a low risk of bias and two out of these five a high level of therapeutic validity. Only two out of the eight studies were indicated as having both a low risk of bias and a high therapeutic validity [18,22]. Both studies showed a significant improvement of HRR after cardiac rehabilitation (CR) compared to controls (absolute between group HRR difference resp. 9 and 5 bpm). 

### Correlation between training intensity and duration and HRR

Linear regression analysis showed no correlation (R^2^) between training intensity, duration and total volume (duration x intensity) and the improvement of the HRR when each selected paper was taken as one data point. This applies to both absolute improvement of HRR and relative improvement (R^2^ all < 0.2 and p > 0.10).

## Discussion

The present systematic review shows that aerobic training increases heart rate recovery in patients with established heart disease. Of the eight eligible studies found in our systematic review, five met the criteria for methodological quality and three met the requirements for therapeutic validity. Only two studies showed both a low risk of bias and a high therapeutic validity. Both studies showed a significant improvement of HRR after exercise training compared to no training. Although only two out of the eight eligible studies met the predetermined criteria and the total number of patients is limited, they showed a homogenic conclusion. On this basis we conclude that the present systematic review shows a level 1A evidence that aerobic training increases heart rate recovery in patients with established heart disease.

HRR has been identified as a powerful predictor of overall mortality [11]. Cole et al. described an adjusted relative risk of 2,0 (95% CI 1.5 - 2.7) for patients with a reduction in heart rate of 12 beats per minute or less after maximal exercise when adjustments were made for age, sex, the use or non-use of medications, the presence or absence of myocardial perfusion defects on thallium scintigraphy, standard cardiac risk factors, the resting heart rate, the change in heart rate during exercise, and workload achieved. Several studies were started thereafter in an attempt to increase HRR in patients with heart diseases using aerobic training, but a systematic review to elucidate the real effects was lacking to our knowledge. 

Exercise-based cardiac rehabilitation has proven its value in reducing total and cardiovascular mortality and hospital admissions with reported relative risk of 0.87 (95% CI 0.75 - 0.99), 0.74 (95% CI 0.63 - 0.87) and 0.69 (95% CI 0.51 - 0.93) respectively [1]. CR is widely recommended for all patients with an acute coronary syndrome (ACS), and for those who have undergone coronary artery bypass graft or valvular surgery or even percutaneous coronary interventions (PCI) [26,27]. Cardio pulmonary exercise testing is considered the gold standard for the measurement of exercise capacity in CR. However, it is expensive, time consuming and not always available for all clinics. In contrast, HRR determination is easy to use and inexpensive. When validity and reliability are determined to be sufficient, it may be a powerful and convenient instrument to monitor improvement in training status of patients with established heart disease.

The observation of HRR increase during exercise based CR in cardiac patients is in line with observations in athletes [28]. Daanen et al. conclude that HRR has the potential to become a valuable tool to monitor changes in training status in athletes and less well-trained subjects. 

Whether the improvement of HRR by CR also reduces mortality is an important question linked to the validity of HRR monitoring. Jolly et al. not only showed in a retrospective cohort study that HRR improved after CR [29], but also found a strong association between abnormal HRR (HRR values ≤ 12 bpm) at exit of CR with all-cause mortality (hazard ratio, 2.15; 95% confidence interval 1.43–3.25). Patients with abnormal HRR at baseline who normalized HRR with exercise had a mortality similar to that of individuals with baseline normal HRR. They evaluated 1070 patients who underwent exercise stress testing before and after completion a CR program. Of 544 patients with abnormal baseline HRR, 225 (41%) had normal HRR after rehabilitation. Among patients with an abnormal HRR at baseline, failure to normalize after rehabilitation predicted a higher mortality (*P* < 0.001).

Despite these clear results, other variables than training status have to be considered as possible confounders for the change in HRR during CR programs. Namely.

All patients were diagnosed as having some kind of cardiovascular disease or surgery. Normal physical recovery after cardiac events (AMI, CAD or revascularisation surgery (CABG or PCI) also influence HRR. During normal recovery from surgery HRR will also increase without an exercise intervention in due time [22]. Therefore, the only way to show the additional effect of aerobic training over normal recovery is to have an experimental and control group. Our review showed an additional effect of exercise training compared to no training or usual care in cardiovascular populations. In all studies the training protocols were clearly defined. The usual care or no training protocol, however, showed some variations. Moholdt et al. presented for instance that the exercise intensity of 'usual care' was described vigorous [24]. 

Secondly HRR is measured over different time frames, generally ranging between 30 seconds and 2 minutes. Most studies use the difference between the end value of exercise and the heart rate after 60 seconds of recovery from an exercise test. In addition to this variation, the exercise intensity during the recovery period is not always described in detail. This can vary from complete rest to a percentage of maximal exercise intensity. In order to be able to compare HRR results, consensus should be reached regarding the way the recovery period is organized. We propose, in line with most studies, to have no exercise at all.

Finally the type of exercise modality can be of influence on HRR. HRR yields higher values for running than for cycling, which is probably related to the higher aerobic demands in running [30]. Although our review consisted of bicycle and treadmill exercise tests, both studies with a low risk of bias and a high therapeutic validity were executed on a bicycle. It can be expected that the relation between training status and HRR is better for treadmill testing than cycling. 

While a clear effect exists of exercise therapy per se, no correlation was found between the duration or intensity of exercise therapy and the change in HRR when every included study was taken as a data point. This is probably due to the considerable differences between the studies in terms of training duration, training intensity, type of exercise, patient group included and so on.

An interesting question remains if the results of the present review can be generalized to other cardiac patients or other diseases. We speculate that the benefits of exercise training would extend to these patient groups, although further research will be needed to demonstrate this. For patients with established heart disease HRR has a prognostic value and can be improved by cardiac rehabilitation.

## Supporting Information

Checklist S1 (DOC)

Table S1 **Full bibliography of the electronic searches.**
(DOCX)

Table S2 **Assessment of risk of bias scale.**
(DOCX)

Table S3 **Overview of studies not included in the review.**
(DOCX)
