# Supplementary material for: Effect of Aerobic Training on Heart Rate Recovery in Patients with Established Heart Disease; a Systematic Review
Source: PLoS One. 2013 Dec 18;8(12):e83907. doi: 10.1371/journal.pone.0083907 (PMC3867471; doi:10.1371/journal.pone.0083907)
Supplement: Table S1 — Full bibliography of the electronic searches. (DOCX) [file pone.0083907.s002.docx]

**Table S1.** Full bibliography of the electronic searches.

#### Search History PUBMED

| **ID** | **SEARCH** | **HITS** |
| --- | --- | --- |
| #4 | Exercise OR Training AND "Heart Rate Recovery" AND "Heart Disease" | **32** |
| #3 | "Heart Disease" | 124.047 |
| #2 | "Heart Rate Recovery" | 466 |
| #1 | Exercise OR Training | 1.162.371 |

#### Search string Embase

| **ID** | **SEARCH** | **HITS** |
| --- | --- | --- |
| #7 | 'heart rate recovery' AND ('heart disease'/exp OR 'heart disease') AND (('exercise'/exp OR exercise) OR ('training'/exp OR training)) | **282** |
| #5 | ('exercise'/exp OR exercise) OR ('training'/exp OR training) | 716.551 |
| #4 | 'heart disease'/exp OR 'heart disease' | 1.250.783 |
| #3 | 'heart rate recovery' | 679 |
| #2 | 'training'/exp OR training | 434.901 |
| #1 | 'exercise'/exp OR exercise | 338.532 |

#### Search string Central (Cochrane Clinical Trials)

| **ID** | **SEARCH** | **HITS** |
| --- | --- | --- |
| #1 | Heart Rate Recovery | **5** |

**Search string Scopus**

| **ID** | **Search** | **hits** |
| --- | --- | --- |
| #4 | "Heart Rate Recovery" AND Exercise OR Training AND "Heart Disease" | **65** |
| #3 | "Heart Disease" | 282.261 |
| #2 | exercise OR training | 848.263 |
| #1 | "Heart Rate Recovery" | 259 |
