# Supplementary material for: Effect of Aerobic Training on Heart Rate Recovery in Patients with Established Heart Disease; a Systematic Review
Source: PLoS One. 2013 Dec 18;8(12):e83907. doi: 10.1371/journal.pone.0083907 (PMC3867471; doi:10.1371/journal.pone.0083907)
Supplement: Table S2 — Assessment of risk of bias scale. (DOCX) [file pone.0083907.s003.docx]

**Table S2.** Assessment of risk of bias scale.

| **Items** | | | | **Judgment** | | | | |
| --- | --- | --- | --- | --- | --- | --- | --- | --- |
| A) Sequence generation | | | |  | |  | |  |
| 1 | Was the method of randomization adequate? | | Yes | | No | | Unsure | |
|  | A random (unpredictable) assignment sequence. Examples of adequate methods are coin toss (for studies with two groups), rolling a dice (for studies with two or more groups), drawing of balls of different colours, drawing of ballots with the study group labels from a dark bag, computer-generated random sequence, pre-ordered sealed envelopes, sequentially-ordered vials, telephone call to a central office, and pre-ordered list of treatment assignments Examples of inadequate methods are: alternation, birth date, social insurance/security number, date invited to participate in the study, hospital registration number, etc.. | | | | | | | |
|  |  | | | | | | | |
| B) Allocation concealment | | | |  | |  | |  |
| 2 | Was the treatment allocation concealed? | | Yes | | No | | Unsure | |
|  | Assignment generated by an independent person not responsible for determining the eligibility of the patients. This person has no information about the persons included in the trial and has no influence on the assignment sequence or on the decision about eligibility of the patient. | | | | | | | |
|  |  | | | | | | | |
| C) Blinding of participants, personnel and outcome | | | |  | |  | |  |
| 3 | Was the patient blinded to the intervention? | | | Yes | | No | | Unsure |
|  | This item should be scored “yes” if the index and control groups are indistinguishable for the patients or if the success of blinding was tested among the patients and it was successful. | | | | | | | |
| 4 | Was the care provider blinded to the intervention? | | | Yes | | No | | Unsure |
|  | This item should be scored “yes” if the index and control groups are indistinguishable for the care providers or if the success of blinding was tested among the care providers and it was successful. | | | | | | | |
| 5 | Was the outcome assessor blinded to the intervention? | | | Yes | | No | | Unsure |
|  | Adequacy of blinding should be assessed for the primary outcomes. This item should be scored “yes” if the success of blinding was tested among the outcome assessors and it was successful or:   - *For patient-reported outcomes* in which the patient is the outcome assessor (e.g., pain, disability): the blinding procedure is adequate for outcome assessors if participant blinding is scored “yes”. - *For outcome criteria assessed during scheduled visit and that supposes a contact between participants and outcome assessors* (e.g., clinical examination): the blinding procedure is adequate if patients are blinded, and the treatment or adverse effects of the treatment cannot be noticed during clinical examination. - *For outcome criteria that are clinical or therapeutic events* that will be determined by the interaction between patients and care providers (e.g., co-interventions, length of stay, treatment failure), in which the care provider is the outcome assessor: the blinding procedure is adequate for outcome assessors if item “E” is scored “yes”. - *For outcome criteria that are assessed from data of the medical forms*: the blinding procedure is adequate if the treatment or adverse effects of the treatment cannot be noticed on the extracted data | | | | | | | |
|  |  | | | | | | | |
| D) Incomplete outcome data | | | |  | |  | |  |
| 6 | Was the drop-out rate described and acceptable? | | | Yes | | No | | Unsure |
|  | The number of participants who were included in the study but did not complete the observation period or were not included in the analysis must be described and reasons given. If the percentage of withdrawals and drop-outs does not exceed 20% for during follow-up and does not lead to substantial bias a ‘yes’ is scored. (N.B. these percentages are arbitrary, not supported by literature). | | | | | | | |
| 7 | Were all randomized participants analysed in the group to which they were allocated? | | | Yes | | No | | Unsure |
|  | All randomized patients are reported/analysed in the group they were allocated to by randomization for the most important moments of effect measurement (minus missing values) irrespective of non-compliance and co-interventions. | | | | | | | |
|  |  | | | | | | | |
| E) Other sources of potential bias | | | |  | |  | |  |
| 8 | | Were the groups similar at baseline regarding the most important prognostic indicators? | | Yes | | No | | Unsure |
|  | | In order to receive a “yes”, groups have to be similar at baseline regarding demographic factors, severity of complaints, and value of main outcome measure(s). | | | | | | |
| 9 | | Were co-interventions avoided or similar? | | Yes | | No | | Unsure |
|  | | This item should be scored “yes” if there were no co-interventions or they were similar between the index and control groups. | | | | | | |
| 10 | | Was the compliance acceptable in all groups? | | Yes | | No | | Unsure |
|  | | The reviewer determines if the compliance with the interventions is acceptable, based on the reported intensity, duration, number and frequency of sessions for both the index intervention and control intervention(s). For example, physiotherapy treatment is usually administered over several sessions; therefore it is necessary to assess how many sessions each patient attended. For single-session interventions (for ex: surgery), this item is irrelevant. | | | | | | |
| 11 | | Was the timing of the outcome assessment similar in all groups? | | Yes | | No | | Unsure |
|  | | Timing of outcome assessment should be identical for all intervention groups and for all important outcome assessments. | | | | | | |
