# Supplementary material for: Effect of Aerobic Training on Heart Rate Recovery in Patients with Established Heart Disease; a Systematic Review
Source: PLoS One. 2013 Dec 18;8(12):e83907. doi: 10.1371/journal.pone.0083907 (PMC3867471; doi:10.1371/journal.pone.0083907)
Supplement: Table S3 — Overview of studies not included in the review. (DOCX) [file pone.0083907.s004.docx]

**Table S3 Overview of studies not included in the Review**

| **Author** | **Study population** | **Design** | **No participants per group** | **Length of rehabilitation** | **Effect HRR**  **60 sec** |
| --- | --- | --- | --- | --- | --- |
| Adams *et al* (2008) | CVD | Ret. Cohort | 210 | 12 wk | 15 🡪 18 |
| Berent *et al* (2010) | CVD | Ret. Cohort | 570 | 3 wk | 15 🡪 19 |
| Dimopoulos *et al* (2006) | CHF | RCT | 14,10 | 12 wk | CT: 15 🡪 24  IT: 21 🡪 21 |
| Giallauria *et al* (2006a) | AMI | Pros. Cohort | 104,164 | 12 wk | ET: 14 🡪 19  C: 14 🡪 14 |
| Giallauria *et al* (2006b) | AMI | RCT | 22,22 | 12 wk | ET: 17 🡪 23  C: 19 🡪 24 |
| Hai *et al* (2010) | CVD | Pros. Cohort | 334,52 | 8 wk | ET: 18 🡪 19  C: 19 🡪 21 |
| Hao *et al* (2002) | CVD | Ret. Cohort | 55 | 12 wk | 15 🡪 19 |
| Jolly *et al* (2011) | CVD | Ret. Cohort | 1070 | 12 wk | 13 🡪 17 |
| Kligfield *et al* (2003) | CVD | Pros. Cohort | 81 | 12 wk | 13 🡪 16 |
| MacMillan *et al* (2006) | CVD | Ret. Cohort | 100 | 12 wk | 21 🡪 24 |
| Piotrowicz *et al* (2009) | CHF | Pros. Cohort | 41 | 8 wk | 12 🡪 13 |
| Roberts *et al* (2006) | CVD | Pros. Cohort | 38 | 8 wk | 7 🡪 9 |
| Sato *et al* (2004) | CABG | Ret. Cohort | 20 | 2 wk | ? |
| Soleimani *et al* (2009) | CVD | Ret. Cohort | 37,168^1^ | 8 wk | DM: 6 🡪 24  nDM: 7 🡪 30 |
| Streuber *et al* (2006) | CHF | Ret. Cohort | 11,18,17 | 12 wk | nTE: 24 🡪 19  TE < 12: 7 🡪 12  TE > 12: 21 🡪 16 |
| Tiukinhoy *et al* (2003) | CVD | Ret. Cohort | 34,35 | 12 wk | ET: 18 🡪 22  C: 21 🡪 21 |
| Tsarouhas *et al* (2010) | CHF | ? | 18 | 12 wk | ET: 12 🡪 17  C: 12 🡪 12 |

1 Data only of male participants. Abbreviations: CABG (Coronary Artery Bypass Graft) HRR (Heart Rate Recovery) CT (Continuous training) IT (Interval training) ET (Exercise training) C (Control group) nTE (No Training Effect) TE < 12 (Training effect and start HRR lower than 12 ) TE > 12 (Training effect and start HRR more than 12) DM (Diabetes Mellitus) nDM (no Diabetes Mellitus).
